# Analysis of influenza data generated by four epidemiological surveillance laboratories in Mexico, 2010–2016

**DOI:** 10.1017/S0950268819000694

**Published:** 2019-05-02

**Authors:** L. Fernandes-Matano, I. E. Monroy-Muñoz, M. Bermúdez de León, Y. A. Leal-Herrera, I. D. Palomec-Nava, J. A. Ruíz-Pacheco, B. L. Escobedo-Guajardo, C. Marín-Budip, C. E. Santacruz-Tinoco, J. González-Ibarra, C. R. González-Bonilla, J. E. Muñoz-Medina

**Affiliations:** 1 Laboratorio Central de Epidemiología, División de Laboratorios de Vigilancia e Investigación Epidemiológica, IMSS, Ciudad de México, Mexico; 2 Escuela Nacional de Ciencias Biológicas, IPN, Ciudad de México, Mexico; 3 Laboratorio de Genómica, Departamento de Genética y Genómica Humana, Instituto Nacional de Perinatología ‘Isidro Espinosa de los Reyes’, Ciudad de México, Mexico; 4 Departamento de Biología Molecular, Centro de Investigación Biomédica del Noreste IMSS, Monterrey, N.L., Mexico; 5 Departamento de Ciencias Básicas, Vicerrectoria de Ciencias de la Salud, Universidad de Monterrey, Av. Ignacio Morones Prieto 4500 Pte., 66238, San Pedro Garza García, N.L., Mexico; 6 Unidad de Investigación Médica Yucatán, Unidad Médica de Alta Especialidad, Centro Médico Nacional ‘Ignacio García Téllez’ IMSS, Mérida, Yucatán, Mexico; 7 Laboratorio de Apoyo a la Vigilancia Epidemiológica (LAVE), Unidad Médica de Alta Especialidad, CMN ‘Ignacio García Téllez’ IMSS, Mérida, Yucatán, Mexico; 8 Cátedra CONACyT, División de Investigación Quirúrgica, Centro de Investigación Biomédica de Occidente IMSS, Guadalajara, Jal., Mexico; 9 Laboratorio de Diagnóstico Molecular Departamento de Biología Molecular, Centro de Investigación Biomédica del Noreste IMSS, Monterrey, N.L., Mexico; 10 División de Laboratorios de Vigilancia e Investigación Epidemiológica, IMSS, Ciudad de México, Mexico

**Keywords:** Infectious disease epidemiology, influenza, molecular biology

## Abstract

The disease caused by the influenza virus is a global public health problem due to its high rates of morbidity and mortality. Thus, analysis of the information generated by epidemiological surveillance systems has vital importance for health decision making. A retrospective analysis was performed using data generated by the four molecular diagnostic laboratories of the Mexican Social Security Institute between 2010 and 2016. Demographics, influenza positivity, seasonality, treatment choices and vaccination status analyses were performed for the vaccine according to its composition for each season. In all cases, both the different influenza subtypes and different age groups were considered separately. The circulation of A/H1N1pdm09 (48.7%), influenza A/H3N2 (21.1%), influenza B (12.6%), influenza A not subtyped (11%) and influenza A/H1N1 (6.6%) exhibited well-defined annual seasonality between November and March, and there were significant increases in the number of cases every 2 years. An inadequate use of oseltamivir was determined in 38% of cases, and the vaccination status in general varied between 12.1 and 18.5% depending on the season. Our results provide current information about influenza in Mexico and demonstrate the need to update both operational case definitions and medical practice guidelines to reduce the inappropriate use of antibiotics and antivirals.

## Introduction

Influenza infections are a public health problem worldwide due to their high morbidity and mortality. The disease can affect people of any age, but it is more common in children [1]. The economic impact of this disease is high for both infected persons and public health institutions due to visits to doctors and days of hospital stay when the condition is serious [2, 3].

According to the International Committee on Taxonomy of Viruses (ICTV), influenza viruses belong to the *Orthomyxoviridae* family and include four genera: *Alphainfluenzavirus* (*influenza A virus*), *Betainfluenzavirus* (*influenza B virus*), *Gammainfluenzavirus* (*influenza C virus*) and *Deltainfluenzavirus* (*influenza D virus*) [4]. The viral genome consists of eight negative-sense RNA strands that encode 11–12 viral proteins. Influenza viruses are named according to their two major membrane proteins: haemagglutinin (HA) and neuraminidase (NA), which are also responsible for their antigenic characteristics. Influenza A virus is the most important due to its high pandemic potential (higher mutation and transmission rates and capacity to spread from person to person and even from animals to humans) [5, 6]. Mutations in HA and NA can cause small changes called *antigenic drift*, which can make the virus reach a better physical state, causing an epidemic. However, when different viruses co-infect the same host cell there may be an exchange of segments, called *antigenic shift*, which establishes a high probability of creating viral strains capable of causing potential pandemics, such as those that began in Spain in 1918 (H1N1), Asia in 1957 (H2N2), Hong Kong in 1968 (H3N2) and, more recently, in Mexico in April 2009 (H1N1) [7, 8].

In the last pandemic, the global number of deaths confirmed by laboratory reports by the WHO was 18 631, but new analyses have estimated that it was up to 10 times higher [9]. During the following years, several outbreaks of the new influenza strain (A/H1N1pdm09), as well as the seasonal strains A/H1N1, A/H3N2 and influenza B were registered in Mexico [10, 11].

Therefore, epidemiological virological surveillance is important because it allows for the detection of circulating strains, transmission patterns and identifying the most vulnerable groups in defined geographic areas and for the design of more efficient interventions against future pandemics [12]. Given the above considerations, the objective of this work was to perform an epidemiological analysis using the data generated from 2010 to 2016 by four epidemiological surveillance laboratories of the Instituto Mexicano del Seguro Social to understand the features of the disease caused by influenza viruses in México, with respect to incidence, seasonality, clinical symptoms, prescription of treatments, affected age groups, deaths and vaccination status.

## Methods

### Study design

A retrospective analysis was performed using data generated by four molecular diagnostic laboratories of the Mexican Social Security Institute (Instituto Mexicano del Seguro Social, IMSS). These laboratories are distributed in the states of Nuevo Leon, Jalisco, Yucatan and México city, which receives samples from all over the country.

From January 2010 to November 2016, 77 841 samples from patients of all ages from the entire country were sent to one of the four laboratories of the IMSS Network for the confirmatory diagnosis of influenza by quantitative reverse transcription quantitative polymerase chain reaction (RT-qPCR) [13]. According to the guidelines of the General Directorate of Epidemiology, the samples sent represent 10% of outpatient cases and 100% of hospitalised cases. Of this total, 55 320 samples were selected that had complete clinical information available in the National System for Epidemiological Surveillance (SINAVE) database and who met one of the following case definitions, according to the Guidelines for Epidemiological Surveillance of Influenza by Laboratory [13]:

Influenza-like illness (ILI): Person of any age who has a fever greater than or equal to 38 °C, cough and headache accompanied by one or more of the following symptoms: runny nose, arthralgia, myalgia, prostration, odynophagia, chest pain, abdominal pain or nasal congestion. In children under 5 years of age, a cardinal sign is irritability, which replaces the headache.

Severe acute respiratory infection (SARI): A person of any age who presents with difficulty breathing accompanied by a fever greater than or equal to 38 °C and a cough with one or more of the following symptoms: effects on general condition, chest pain, polypnoea or acute respiratory distress syndrome, or anyone whose death is associated with SARI. In the case of immunosuppressed patients and those older than 65 years old, fever is not required as a cardinal symptom.

The data were grouped by age groups according to the IMSS on their health cards as follows: 0–9 years, 10–19 years, 20–59 years and 60 years and over. In this way, demographic analysis, positivity, symptomatology, seasonality, choice of treatment and vaccination status (percentage of positive cases with vaccination history in each season) were carried out. The symptomatology was evaluated by determining 18 symptoms, among which irritability was considered a cardinal sign only in children under 5 years of age.

### Laboratory confirmation (RT-qPCR)

Laboratory confirmation was performed following the protocol described by the Centers for Disease Control and Prevention (CDC) [14] for the confirmatory diagnosis of influenza A/H1Npdm09, A/H1N1, A/H3N2 and influenza B. Pharyngeal swabs were taken with Dacron swabs and preserved in viral transport medium (Becton Dickinson 7 Loveton Circle Sparks, USA, Cat: 220220) at 4 °C prior to use. To perform the virus determination, nucleic acids were extracted from 200 µL of the sample using the MagNa Pure LC Total Nucleic Acid Isolation Kit automated system (Roche Diagnostics, Mannheim, Germany; catalogue: 03038505001). The amplification was performed with the SuperScript III Platinum One-step qRT-PCR System (Invitrogen Carlsbad, California, EUA; cat: 12574035) in the 7500 Fast Real-Time PCR System (Applied Biosystems Foster City, California, EUA).

### Statistical analysis

Descriptive statistics were used to analyse the prevalence of the viruses included in the study, and percentages were given with a 95% confidence interval. The *χ*^2^ tests of homogeneity and independence were used to compare categorical variables (*P* < 0.05 values were taken as significant). To analyse the hypothesis of quantitative variables, analysis of variance and Student's *t* tests were used. The analyses were performed using the IBM SPSS Statistics 24.0 program, and the graphs were generated using the Microsoft Excel 2010 program.

## Ethics statement

The information about the biological specimens used in the current study is not traceable to individual patients' identities. All of the samples were used in an anonymous way.

## Results

### Demographics

The study included data generated from 55 320 samples. The population was composed of 48.4% males and 51.6% females. The patient's ages ranged from 0 to 108 years. The population was divided into four age groups as follows: 0–9 years, 10–19 years, 20–59 years and ⩾60 years. The age group with the highest number of cases based on the case definitions during the period covered by the study was the 20–59 year olds with 49.8%, followed by the 0–9 year olds, ⩾60 year olds and lastly the 10–19 year olds, with rates of 23.1, 21.1 and 6.0%, respectively. With regards to the case severity, the percentage of hospitalisations (64.4%) surpassed the outpatient cases (35.6%) (Table 1).

**Table 1.** Demographic data for the samples included in the study

Demographic data		
Gender	*n*	%
Male	26 762	48.4
Female	28 558	51.6
Age group (years)		
0–9	12 786	23.1
10–19	3296	6.0
20–59	27 556	49.8
⩾60	11 682	21.1
Zone		
North	11 666	21.1
Central	29 581	53.5
South	14 073	25.4
Clinical situation		
Hospitalised	35 643	64.4
Ambulatory	19 677	35.6

Regarding the geographic distribution, we obtained a representation of all Mexican territories that was distributed as follows: 21.1% in the north region (Baja California, Baja California Sur, Sonora, Chihuahua, Coahuila, Nuevo León, Durango, Sinaloa, Zacatecas, San Luis Potosí, Tamaulipas, Nayarit and Aguascalientes), 53.5% in the centre (Guanajuato, Queretaro, Hidalgo, Morelos, Jalisco, Colima, Michoacán, Mexico State, Tlaxcala and Distrito Federal) and 25.4% in Puebla, Guerrero, Veracruz, Oaxaca, Tabasco, Chiapas, Campeche, Yucatan and Quintana Roo (Table 1).

### Positivity

**Fig. 1.**
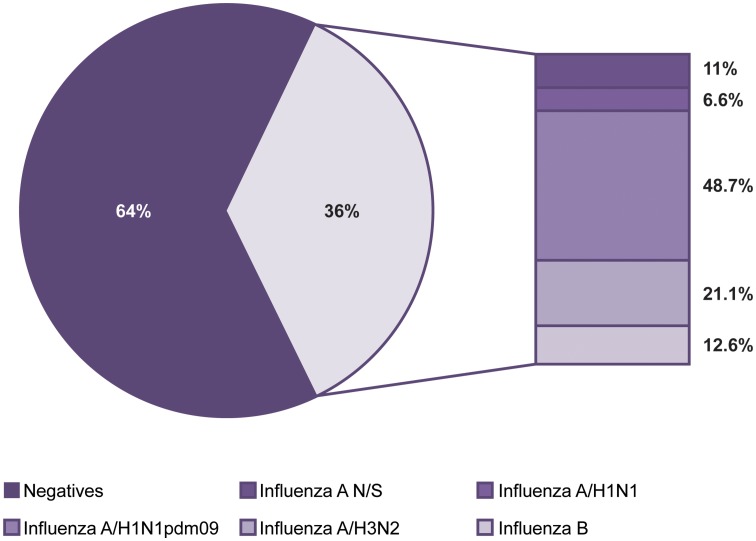
Influenza positivity from 2010 to 2016. The figure shows the positivity observed and the influenza subtypes identified during the study period.

Of the 55 320 samples analysed, 19 725 (36%) were positive and 35 595 (64%) were negative for influenza viruses (Fig. 1). The most frequent subtype was A/H1N1pdm09, which represented 48.7% (9605) of the positive samples, followed by influenza A/H3N2 with 21.1% (4159), influenza B with 12.6% (2483), influenza A not subtyped (influenza N/S) with 11% (2166) and influenza A/H1N1 with 6.6% (1312).

### Seasonality

**Fig. 2.**
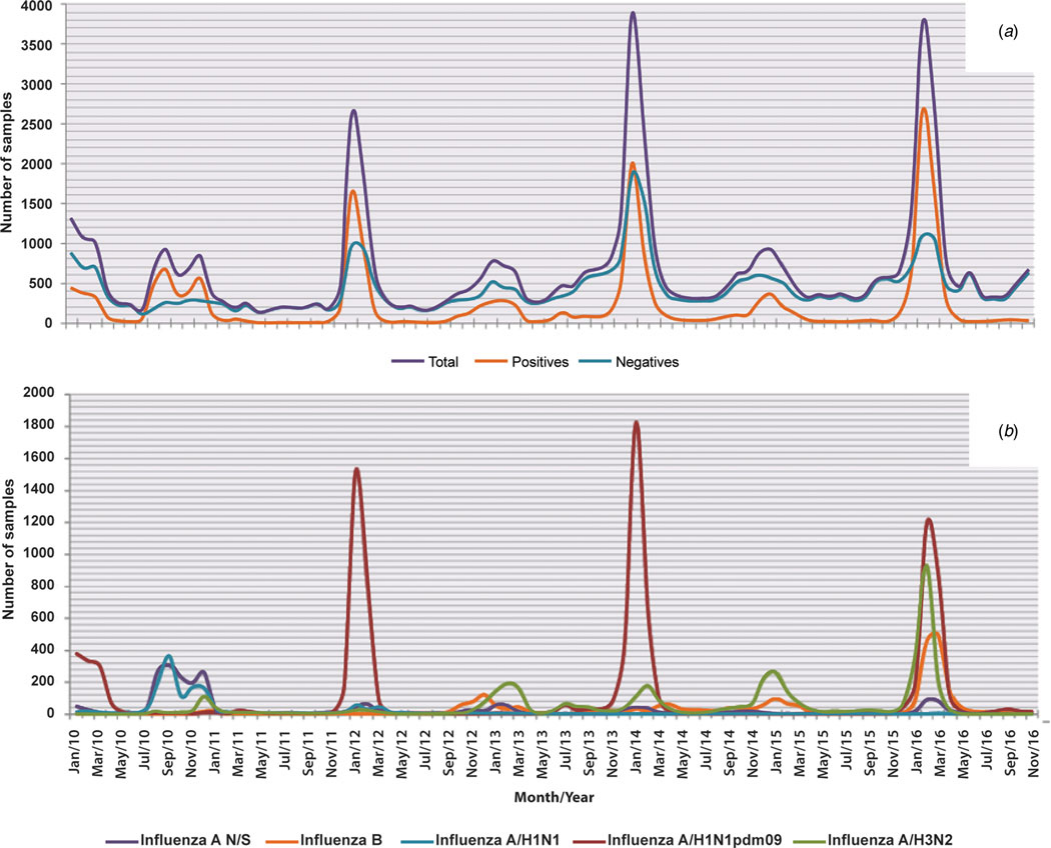
Seasonality of influenza and negative cases from 2010 to 2016. The figure shows the monthly circulation of influenza, the negative cases and each subtype identified during the study period (2010–2016). (a) Total, positive and negative cases of influenza and (b) influenza subtypes.

The influenza cases presented seasonality with a single peak between the months of November and March of each year (*P* < 0.05). We also observed that both the positivity within these months and the number of cases increased significantly every 2 years, corresponding to the 2010, 2012, 2014 and 2016 seasons. With the exception of the mentioned peaks, negative cases were more abundant throughout the year (Fig. 2a). With regards to influenza, although the occurrence of cases in general was constant in terms of numbers and dates, the subtypes identified in each season varied unpredictably. However, cases caused by subtype A/H3N2 are those that can be observed more regularly, with annual peaks as shown in Figure 2b. Also in this figure, it can be clearly perceived that in 2016, there was a decrease in the circulation of subtype A/H1N1pdm09 and an increase in circulation of the other subtypes, with the exception of A/H1N1 that has not been detected since 2012 (Fig. 2b).

### Analysis by age group

**Fig. 3.**
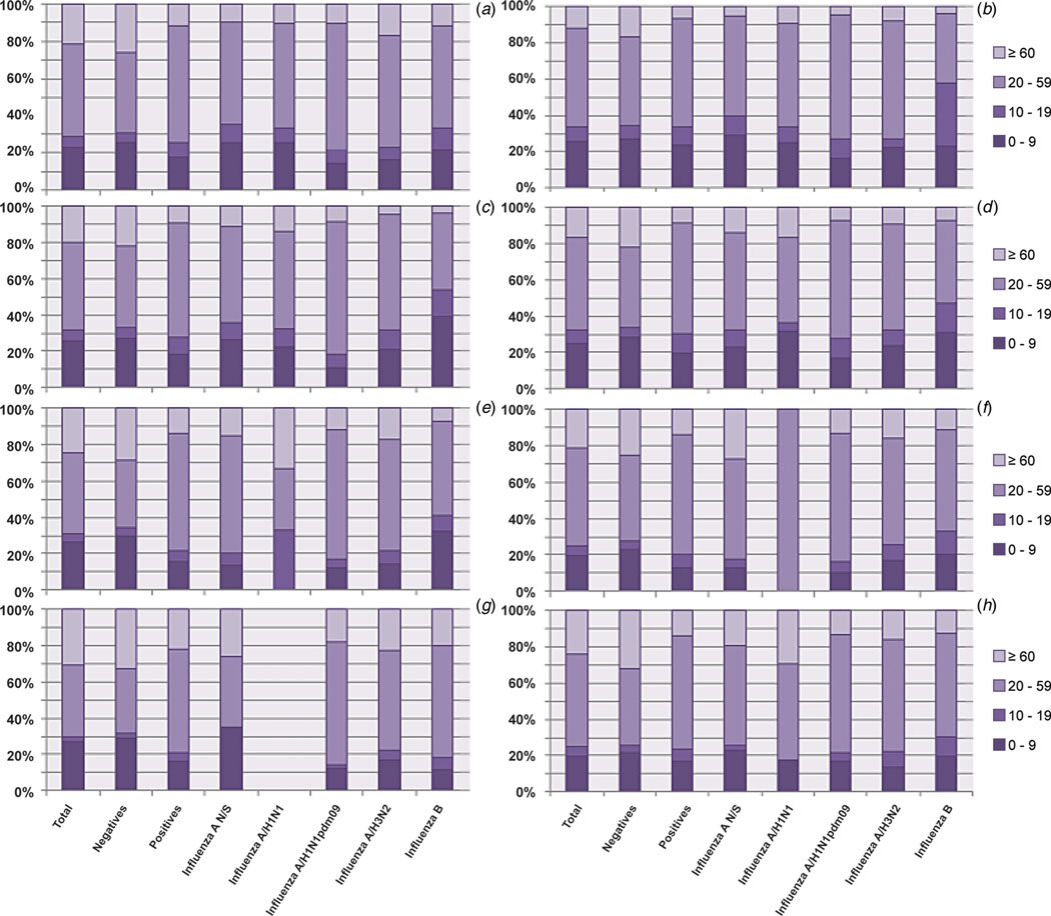
Analysis of the proportion of different subtypes in each age group from 2010 to 2016. The figure shows the proportion of influenza cases in general and by subtype in each age group throughout the study period. (a) Total; (b) 2010; (c) 2011; (d) 2012; (e) 2013; (f) 2014; (g) 2015 and (h) 2016.

Analysis of the influenza subtypes by age group revealed that the age group with the highest proportion of positive cases regardless of the viral subtype was the 20–59 year olds, followed by the 0–9 year olds, the ⩾60 year olds and finally the 10–19 year olds, as shown in Figure 3a. Although small variations can be observed, this tendency was maintained during all seasons (Fig. 3b–h).

### Clinical situation and severity

To determine the severity of the infections caused by the different influenza subtypes, we evaluated the symptomatology presented in each case, the average number of symptoms observed, the percentage of patients requiring hospitalisation and the number of deaths reported (Table 2).

**Table 2.** Symptomatology, mortality rate and hospitalisation by influenza strain

Symptom	Total *n* (%)	Negatives *n* (%)	Positives *n* (%)	A N/S *n* (%)	Seasonal A/H1N1 *n* (%)	A/ H1N1pdm09 *n* (%)	Seasonal A/H3N2 *n* (%)	Inf B *n* (%)
Fever	40 031 (72.4)	24 478 (68.8)	15 553 (78.8)	1772 (81.8)	931 (71.0)	7600 (79.1)	3296 (79.2)	1954 (78.7)
Cough	50 275 (90.9)	32 006 (89.9)	18 269 (92.6)	1923 (88.8)	1211 (92.3)	8901 (92.7)	3890 (93.5)	2344 (94.4)
Headache	40 075 (72.4)	23 858 (67.0)	16 217 (82.2)	1722 (79.5)	1027 (78.3)	7873 (82.0)	3477 (83.6)	2118 (85.3)
Odynophagia	28 210 (51.0)	16 845 (47.3)	11 365 (57.6)	1268 (58.5)	821 (62.6)	5407 (56.3)	2388 (57.4)	1481 (59.6)
Myalgias	34 483 (62.3)	20 099 (56.5)	14 384 (72.9)	1503 (69.4)	900 (68.6)	7054 (73.4)	3075 (73.9)	1852 (74.6)
Arthralgias	31 310 (56.6)	18 308 (51.4)	13 002 (65.9)	1396 (64.5)	845 (64.4)	6413 (66.8)	2721 (65.4)	1627 (65.5)
Prostration	27 342 (49.4)	18 137 (51.0)	9205 (46.7)	1031 (47.6)	537 (40.9)	4666 (48.6)	1868 (44.9)	1103 (44.4)
Rhinorrhoea	36 238 (65.5)	21 616 (60.7)	14 622 (74.1)	1699 (78.4)	1089 (83.0)	6785 (70.6)	3136 (75.4)	1913 (77.0)
Chills	32 650 (59.0)	19 399 (54.5)	13 251 (67.2)	1387 (64.0)	844 (64.3)	6525 (67.9)	2835 (68.2)	1660 (66.9)
Abdominal pain	13 884 (25.1)	8399 (23.6)	5485 (27.8)	622 (28.7)	432 (32.9)	2676 (27.9)	1073 (25.8)	682 (27.5)
Conjunctivitis	13 122 (23.7)	7474 (21.0)	5648 (28.6)	649 (30.0)	419 (31.9)	2628 (27.4)	1220 (29.3)	732 (29.5)
Dyspnoea	33 842 (61.2)	24 175 (67.9)	9667 (49.0)	800 (36.9)	497 (37.9)	5101 (53.1)	2070 (49.8)	1199 (48.3)
Cyanosis	5773 (10.4)	4294 (12.1)	1479 (7.5)	113 (5.2)	75 (5.7)	830 (8.6)	303 (7.3)	158 (6.4)
Diarrhoea	6681 (12.1)	4307 (12.1)	2374 (12.0)	272 (12.6)	145 (11.1)	1203 (12.5)	427 (10.3)	327 (13.2)
Thoracic pain	25 897 (46.8)	16 890 (47.5)	9007 (45.7)	819 (37.8)	497 (36.5)	4742 (49.4)	1881 (45.2)	1086 (43.7)
Polypnoea	5773 (10.4)	4294 (12.1)	1479 (7.5)	113 (5.2)	75 (5.7)	830 (8.6)	303 (7.3)	158 (6.4)
Irritability	8135 (14.7)	6292 (17.7)	1843 (9.3)	237 (10.9)	178 (13.6)	845 (8.8)	353 (8.5)	230 (9.3)
Coryza	8100 (14.6)	4916 (13.8)	3184 (16.1)	260 (12.0)	212 (16.2)	1551 (16.1)	700 (16.8)	461 (18.6)
Average number of symptoms	7.99	7.75	8.34	8.12	8.17	8.50	8.42	8.49
Hospitalised cases	35 643 (64.4)	26 252 (73.8)	9391 (47.6)	743 (34.3)	373 (28.4)	5179 (53.9)	1952 (46.9)	1144 (46.1)
Deaths	3588 (6.5)	2317 (6.5)	1271 (6.4)	65 (3.0)	39 (3.0)	952 (9.9)	136 (3.3)	79 (3.2)

We observed that the average symptoms were higher in the positive samples than in the negative ones (8.34 *vs.* 7.75, *P* < 0.05). A comparison of the average symptoms among the subtypes showed that influenza A/H1N1pdm09, influenza A/H3N2 and influenza B generated more clinical manifestations than influenza A not subtyped and influenza A/H1N1 (*P* < 0.05).

In the positive samples, coughing was the most common symptom and was present in 92.6% of the cases, followed by headache (82.2%) and fever (78.8%). Irritability, a symptom considered only in children under 5 years of age, was significantly higher (*P* < 0.05) in patients with a negative result, and the most serious or debilitating symptoms, such as cyanosis, polypnoea, dyspnoea and prostration, were detected in a significantly higher percentage of the negative samples than in the positive samples (*P* < 0.05) and in infections with the A/H1N1pdm09 strain than in infections with the other influenza strains (*P* < 0.05).

Conversely, arthralgia, myalgia, conjunctivitis, rhinorrhoea and headaches were significantly more frequent in infections caused by the different influenza strains than in the negative samples (*P* < 0.05) (Table 2). In the particular case of rhinorrhoea and conjunctivitis, in the group of positive samples, these symptoms were less commonly associated with influenza A/H1N1pdm09.

**Fig. 4.**
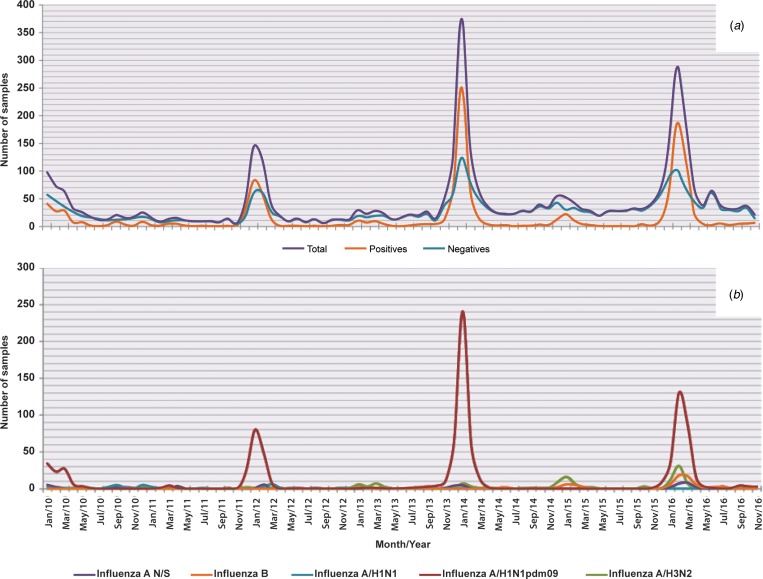
Deaths in general and by influenza subtype from 2010 to 2016. The figure shows monthly deaths in general and by subtype of influenza from 2010 to 2016. (a) In the total sample, in the positive ones and in the negative ones for influenza and (b) in each subtype.

Interestingly, in general, the percentage of hospitalisations was higher in patients with a negative result for influenza (73.8%) than in patients with positive results (47.6%) (*P* < 0.05); however, in the group of patients with positive results, those infected with influenza A/H1N1pdm09 had the highest percentages of hospitalisation (53.9%) and death (9.9%) (*P* < 0.05) (Table 2 and Fig. 4).

Regarding deaths, in Figure 4a, it was observed that outside of the influenza season, there were practically not caused by any of the subtypes of this virus, with some exceptions, such as in the 2013/2014 and 2014/2015 seasons, when there were deaths associated with subtypes A/H3N2 and B (Fig. 4b).

### Prevention and treatment

**Fig. 5.**
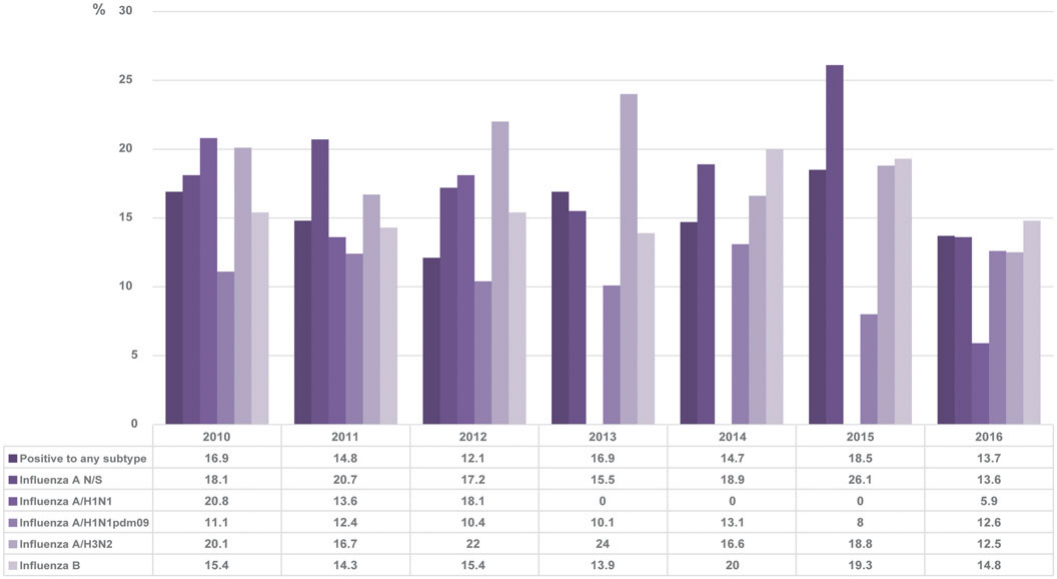
Percentage of positive cases with vaccination history in each season. The figure shows the percentages of positive cases with a history of vaccination for each subtype of influenza and for the positive samples in general.

The results obtained in this study showed that generally, 14.8% of the patients who tested positive by RT-qPCR had a history of receiving the vaccine compared with 16.7% in total. The vaccination status varied between 12.1 and 18.5% without distinguishing between subtypes during the period covered by the study. The percentage of positive patients for each subtype that was vaccinated in each season is shown in Figure 5.

Regarding the type of treatment prescribed, only 31.5% of patients with influenza received the appropriate treatment; only 1.8% of the patients were medicated with antivirals (oseltamivir phosphate) and 29.7% with an antiviral + some antibiotic.

However, antibiotics were prescribed in 36.2% of cases. In addition, we found that oseltamivir phosphate was prescribed in 38.7% of the negative cases (38% of the time prescribed together with an antibiotic, and 0.7% only with an antiviral) (Table 3).

**Table 3.** Relationship between the prescribed treatment and the laboratory result

	Antibiotic *n* (%)	Antiviral *n* (%)	Both *n* (%)	No treatment *n* (%)
Total	598 (1.1)	624 (1.1)	19 392 (35.1)	34 706 (62.7)
Negatives	367 (1.0)	262 (0.7)	13 527 (38.0)	21 439 (60.2)
Positives	141 (0.7)	362 (1.8)	5865 (29.7)	13 357 (67.7)
Influenza A N/S	8 (0.4)	18 (0.8)	434 (20.0)	1706 (78.8)
Influenza A/H1N1	8 (0.6)	18 (1.4)	312 (23.8)	974 (74.2)
Influenza A/H1N1pdm09	125 (1.3)	230 (2.4)	2897 (30.2)	6353 (66.1)
Influenza A/H3N2	0 (0)	63 (1.5)	1386 (33.3)	2710 (65.2)
Influenza B	0 (0)	33 (1.3)	836 (33.7)	1614 (65.0)

We also perceived that the group of negative cases had a lower percentage of untreated patients (with antibiotics, antivirals or both) compared with the group of positive ones (60.2 and 67.7%, respectively). When the analysis was performed by subtype, the patients who were most commonly treated were those infected with influenza A/H3N2 and influenza B (Table 3).

## Discussion

In Mexico, as well as in many other countries, health systems began to pay more attention to the detection of this type of infection and increased their efforts to adhere to international monitoring protocols after the last influenza pandemic in 2009 [15]. In this sense, this study evaluated the time periods in the history of the country in which there was a greater amount of epidemiological data regarding influenza cases. This period began with the 2010 season, which was a year in which the systems were already implemented within the IMSS, and continued to the present (for this study, a cross sectional study was conducted in November 2016). Therefore, the results are highly informative of the real situation in the country.

When analysing the results of all seasons combined, we found that 36% of samples tested were positive for influenza, which was similar to the findings in the study by Kowalczyk *et al*. [16] conducted in Poland during the 2015–2016 season in which a total of 5070 patients over 14 years of age were included in the study, and a positivity of 40.2% was observed; however, the percentage of positivity found in this study was much higher than the percentages reported in other studies worldwide. For instance, Qi *et al*. [17] investigated the period from 2011 to 2015 with 111 589 patients and observed a 13.3% positivity rate, whereas Al-Awaidy *et al*. [18] conducted a study in Oman during the 2008–2013 seasons in 5147 hospitalised children and found only an 8% positivity rate.

We can also see with this work that the negative cases were more abundant throughout the year (except in the peaks of influenza), which indicates a high incidence of other aetiological agents causing respiratory infections. Even so, the results highlight the role of influenza viruses because, although the aetiology of respiratory infections is at least 13 other viruses in Mexico, none of them is present in such a high proportion as influenza [19]. However, the strategies for prevention and treatment should not be directed only against influenza because we cannot neglect the great diversity of viruses that cause these types of infection in the country. This approach should be dynamic and based on current prevalence and incidence data and should be applied by health professionals in both the daily treatment of patients and in health decision-making.

The analysis of seasonality allowed us to determine the presence of cases throughout the year. Significant increases occurred from November to March, which coincided with the dates indicated by the CDC and the Pan American Health Organization (PAHO) for influenza season in the Northern Hemisphere. However, even among countries in this hemisphere, the influenza season does not always occur within well-defined periods of seasonality. An example of this can be found in China, where, in most years, two peaks are observed: one between January and March and another between June and July [17].

This variability in the periods in which influenza cases occur explains in part the discrepancy in the information found in the literature among studies in which the seasonality of the viruses is explained based on climatic conditions (i.e. temperature and humidity). Some studies report that cases increase between November and March, which is a period associated with less rainfall and lower temperatures [20–23]; however, studies have also found that the seasonality of the virus is associated with the rainy season [24].

In Mexico, cases of influenza are presented in a uniform manner both in terms of seasonality and number of cases. For instance, we observed that a significant increase occurred every 2 years. This increase coincided with the appearance of subtype A/H1N1pdm09, which presented the highest percentage of detection in the 2010 (29.6%), 2012 (75.6%), 2014 (66.3%) and 2016 (45.1%) seasons. This fluctuation in the cases caused by the A/H1N1pdm09 strain cannot be explained by changes in the composition of the vaccine because, beginning in 2009, the component for the subtype A/H1N1 has been strain A/California/7/2009 [2]. Thus, the incidence increases or decreases regardless of whether there is a good match between the vaccine and the circulating strain. The opposite case was presented with subtype A/H1N1 (known to be seasonal), as this strain was only present between the 2010 season and the beginning of 2012, after which time it practically disappeared; cases of the strain were not reintroduced until the 2016 season.

In the cases of subtype A/H3N2 and influenza B, the decreases in positivity exactly match the modifications made in the composition of the vaccine [25–30], which shows the positive effect of the choice of strains based on epidemiological surveillance [31] conducted at the level of the Northern Hemisphere.

When these results were analysed together, we observed that in Mexico a very particular situation occurs with respect to the seasonality of influenza cases, as a constant pattern has occurred over the last 6 years, which is characterised by an increase in incidence every 2 years. However, due to aspects such as a great variety of subtypes, changes and antigenic drift and pandemic potential, it is difficult to make predictions about the seasonality of this virus for the following seasons, as happened in the 2016–2017 season, where this behaviour was not maintained and a greater number of cases was observed than in the 2017–2018 season [32, 33].

In this study, a greater proportion of positivity was observed in the group aged 20–59 years, which represents the most productive sector of the population. Similar results have been observed in other countries, such as China, between 2011 and 2013 [34].

The decrease in the positive cases in the 0–9- and 10–19-year-old age groups reflects the positive results of the vaccination campaigns conducted in the country. These campaigns are more effective in younger populations, such as students and younger children, because the vaccine is applied in schools or during doctor visits.

From another perspective, the observation of low positivity in an age group in which a large number of cases is associated with a clinical picture that is concordant with respiratory infection suggests the presence of other aetiological agents that preferentially affect these sectors of the population. These agents are known to be viruses, such as respiratory syncytial virus, which affects children to a greater extent [35]. However, more studies are needed to identify the most common aetiological agents in the population older than 60 years. These trends were maintained throughout all of the seasons regardless of the viral subtype rotation.

Another finding of this study revealed the presence of other agents that cause acute respiratory infections and circulate with influenza in the country. Analysing the data by the severity of the infection suggests that infections caused by influenza trigger a less acute form of infection with less serious complications than other aetiological agents, since the number of symptoms present, the percentage of patients who require hospitalisation and the number of deaths caused are lower in the group of positive cases compared with the group of negative cases. This finding coincides with the observations reported by Zheng *et al*. [36] in a study conducted in China, which evaluated the severity of the infection and the duration of the disease from hospitalisation to discharge/death.

In our study, although the proportion of deaths was lower in the positive cases, this value continued to be higher than the values reported in other studies, such as that of Kowalczyk *et al*. [16], where 140 people out of 8542 gives a case-fatality rate in 1.63% was observed. In our population, the percentage of deaths reached 4.6% in general and almost 10% in the particular case of subtype A/H1N1pdm09.

Regarding the symptoms of influenza, it is still a problem to try to make an assertive diagnosis based solely on clinical symptoms. In a study conducted in Germany, they found that only 44% of confirmed cases were suspected to be influenza by physicians when they were guided by the definition of ILI [37]. In our work, we observed that some of the symptoms most associated with influenza-positive cases were arthralgias, myalgias, conjunctivitis, fever and headaches, while negative cases were those most associated with cyanosis, polypnoea, dyspnoea and prostration. These symptoms are generally more severe and also stood out in the infections caused by the subtype A/H1N1pdm09. However, between both groups (positive and negative for influenza), there were no significant differences in the presence of cough, diarrhoea or chest pain, so these are not symptoms that would help to differentiate a case caused by influenza from one caused by other possible aetiological agents. When we analysed the data for the subtype A/H1N1pdm09, we perceived that, unlike the other subtypes, it was less likely to be associated with conjunctivitis and rhinorrhoea. Taking into consideration that two operational case definitions are currently being followed to guide the use of diagnostic tests, this type of analysis can be used to create a better operative case definition for influenza or to adjust the definitions currently being used and thus improve decision making with regards to the treatment, since it is more effective when administered at an early stage and its use is recommended even before laboratory confirmation is achieved [38].

Notably, the percentage of negative cases treated with oseltamivir exceeded that of the positive cases, and 30.4% of the patients infected with influenza were treated with antibiotics. This practice has also been reported in other studies, where high percentages of the indiscriminate use of antibiotics have been reported during the treatment of respiratory infections of viral origin [39, 40].

Antibiotics are prescribed for one of two possibilities: the prevention of a secondary bacterial infection or the direct suspicion of a primary bacterial infection. However, what proportion of the prescriptions is appropriate is unknown. Therefore, this topic should be the subject of further investigation, since resistance caused by the inappropriate use of antibiotics is a global problem [36].

Finally, for the vaccination status, we analysed the percentage of patients with a positive result for influenza and a confirmed history of having received the vaccine of the corresponding season. We obtained percentages ranging between 12.1 and 18.5%.

When the same analysis was performed separately for different subtypes, it was perceived that the two with the highest percentages of vaccination status were influenza A not subtyped and influenza A/H3N2. In particular, patients positive for subtype A/H3N2 show higher percentages of a vaccination history compared with any of the other subtypes, although in general, the percentage of vaccinated patients was low, as, for example, in 2012 and 2013. This suggests that the vaccine may be less protective against this subtype compared with others. Even so, in general, our percentages of influenza-positive patients with a history of vaccination are lower than those reported by other studies, as, for example, in the case of influenza A/H3N2, we obtained in 2011 and 2012, 16.7 and 22% in each year, respectively, but a study conducted in Europe showed that between 54.9 and 64% of hospitalised patients positive for this subtype had a history of having received the vaccine in these years, and this demonstrated their low effectiveness [41]. However, it should be borne in mind that the result of this type of analysis depends on many variables, such as the number of vaccinated individuals in general during each season and the basic health status of the subjects.

## Conclusions

This study shows that the influenza season in Mexico presents clear seasonality at well-defined dates with peaks that cannot be predicted based on the subtype of influenza that will circulate but that present predictable magnitudes with respect to the other seasons. Additionally, our results provide current information about the symptomatology of influenza in Mexico and demonstrate the need to update both operational case definitions and medical practice guidelines to reduce the inappropriate use of antibiotics and antivirals.
